# Adaptations to strength training differ between endurance-trained and untrained women

**DOI:** 10.1007/s00421-020-04381-x

**Published:** 2020-05-05

**Authors:** Olav Vikmoen, Truls Raastad, Stian Ellefsen, Bent R. Rønnestad

**Affiliations:** 1 Section for Health and Exercise Physiology, Institute of Public Health and Sport Sciences, Inland Norway University of Applied Sciences, Elverum, Norway; 2 Department of Physical Performance, Norwegian School of Sport Sciences, Ullevål Stadion, P.O.box 4014, 0806 Oslo, Norway; 3 Innlandet Hospital Trust, Lillehammer, Norway

**Keywords:** Concurrent training, Muscle strength, Squat jump, Counter movement jump, Muscle hypertrophy

## Abstract

**Purpose:**

The purpose of this study was to investigate if endurance athletes, sustaining their normal endurance training, experience attenuated adaptations to strength training compared to untrained individuals.

**Methods:**

Eleven non-strength-trained female endurance athletes (*E* + *S*) added 11 weeks of strength training to their normal endurance training (5.1 ± 1.1 h per week), and 10 untrained women (*S*) performed the same strength training without any endurance training. The strength training consisted of four leg exercises [3 × 4 − 10 repetition maximum (RM)], performed twice a week for 11 weeks.

**Results:**

*E* + *S* and *S* displayed similar increases in 1RM one-legged leg press (*E* + *S* 39 ± 19%, *S* 42 ± 17%, *p* < 0.05), maximal isometric torque in knee extension (*E* + *S* 12 ± 11%, *S* 8 ± 10%, *p* < 0.05) and lean mass in the legs (*E* + *S* 3 ± 4%, *S* 3 ± 3%, *p* < 0.05). However, *S* displayed superior increases in peak torque in knee extension at an angular velocity of 240° sec^−1^ (*E* + *S* 8 ± 5%, *S* 15 ± 7%, *p* < 0.05) and maximal squat jump height (*E* + *S* 8 ± 6%, *S* 14 ± 7%, *p* < 0.05).

**Conclusions:**

In this study, concurrent training did not impair the adaptations in the ability to develop force at low contraction velocities or muscle hypertrophy. However, concurrent training attenuated strength training-associated changes in the ability to develop force at higher muscular contraction velocities.

## Introduction

Since the pioneering study by Hickson (1980), showing that performing strength and endurance training in the same training program (concurrent training) can attenuate increases in maximal strength compared to strength training alone, a large numbers of studies have assessed the effects of concurrent training. Many of these studies have confirmed that concurrent training can lead to impaired strength gains (Bell et al. 2000; Cadore et al. 2010; Gergley 2009; Hunter et al. 1987; Izquierdo et al. 2005; Jones et al. 2013), impaired muscle hypertrophy (Bell et al. 2000; de Souza et al. 2007; Gergley 2009; Putman et al. 2004) and reduced neural adaptations (Cadore et al. 2010; Hakkinen et al. 2003) compared to strength training alone. However, there are also studies that report no negative effects of concurrent training on strength- and hypertrophy-related adaptations (Abernethy and Quigley 1993; Cantrell et al. 2014; Gravelle 2000; Holviala et al. 2012; McCarthy et al. 1995; McCarthy et al. 2002; Nelson et al. 1990; Shaw et al. 2009). These conflicting data can probably be ascribed differences in the methodological approaches related to aspects such as training status of the participants, training modality, frequency and volume of strength and endurance training, the manner of integration of the two types of training and the selection of dependent variables.

Interestingly, it has been reported that concurrent training attenuates improvements in peak force at high contraction velocities (Dudley and Djamil 1985), jumping performance (Glowacki et al. 2004; Hunter et al. 1987) and maximal rate of force development (RFD) (Hakkinen et al. 2003) compared to strength training only, despite similar increases in isometric force or one repetition maximum (1RM). This indicates that improvements in the ability to produce force and power at high contraction velocities are more attenuated by concurrent training than changes in the ability to produce high forces during slow shortening velocities. On the contrary, endurance performance seems to be improved in endurance athletes that add heavy strength training to their normal endurance training (e.g. Aagaard et al. 2011; Ronnestad et al. 2015; Sedano et al. 2013), and anecdotally the use of strength training among endurance athletes is increasing. However, the typical studies investigating the concurrent training effect include three groups of similarly trained individuals; one group performing strength training only, one group performing endurance training only and one group performing both the strength training and the endurance training. This leaves the concurrent group with a substantially higher increase in total training volume, prompting it for surplus changes in performance. A possible solution to this issue would be to add strength training to the already ongoing endurance training protocol of endurance athletes, generally finding themselves at a steady-state training and performance level, and to compare its effects with those of adding strength training to previously untrained individuals. This set-up ensures similar changes in training volume in the two groups.

To the best of our knowledge, only two studies have investigated the effects of concurrent training using such a design (Hunter et al. 1987; Ronnestad et al. 2012), though with conflicting conclusions. Whereas Hunter et al. (1987) reported similar increases in 1RM squat and counter movement jump (CMJ) in runners and previously untrained individuals after performing strength training, Ronnestad et al. (2012) reported attenuated gains in 1RM leg strength, thigh muscle CSA, maximal RFD and squat jump (SJ) height in highly trained male cyclists. This discrepancy may be due to the difference in the volume of endurance training. Hunter et al. (1987) assessed recreational active runners performing 1–3 h endurance training per week, whereas Ronnestad et al. (2012) assessed competitive cyclists performing about 10 h of endurance training per week. Consequently, there is need for studies that elaborate on the effects of concurrent endurance and strength training and its effect on strength-related variables using this design. Furthermore, this design has not previously been used to investigate the effects of concurrent training in female endurance athletes.

The purpose of this study was to compare the effects of 11 weeks of strength training on changes in lean mass in the legs (leg_LM_), 1RM, isometric torque, maximal torque at high contraction velocities, and jumping performance between well-trained female endurance athletes with no strength training experience that sustains their normal endurance training and previously untrained age-matched women.

## Methods

### Ethical approval

The study was approved by the Local Ethics Committee at Inland Norway University of Applied Sciences. Written informed consent was obtained from all participants prior to inclusion, and the study was carried out in accordance with the Declaration of Helsinki.

### Participants

Fourteen well-trained female endurance athletes that was active in both cycling and running, [classified according to Jeukendrup et al. (2000)], and 10 untrained female participants were recruited to this study. None of the participants had performed systematic strength training for the 12 months leading up to the study. During the study, three of the endurance athletes left the project for reasons unrelated to the project protocol: one because of an injury not related to the strength training, one because of a prolonged period off illness during the last part of the intervention and one because of other medical reasons.

### Experimental overview

The study was part of a larger study, investigating the effects heavy strength training on various aspects of cycling and running performance. Some of the results on endurance performance have been previously reported (Vikmoen et al. 2016a, b, 2017).

The endurance athletes added heavy strength training to their normal endurance training for 11 weeks (*E* + *S*, *n* = 11). The endurance training mainly consisted of cycling and running at an average of 5.1 ± 1.1 h per week [for details see Vikmoen et al. (2016a)]. The untrained participants performed the same strength training program, while habitually performing at most one endurance training session per week in addition to the strength training (*S*, *n* = 10). The characteristics of the participants are shown in Table 1.

**Table 1:** Characteristics of the athletes adding strength training to their normal endurance training (*E* + *S*) and the untrained individuals performing strength training only (*S*)

Group	N	Age (years)	Height (m)	Body mass (kg)	BMI (kg/m^2^)	*V*O_2max_ (ml kg^−1^ min^−1^)
*E* + *S*	11	31.5 ± 8.0	1.69 ± 0.05	62.2 ± 5.2	21.7 ± 1.3	54 ± 3
*S*	10	31.0 ± 9.9	1.72 ± 0.04	67.8 ± 13.5	22.8 ± 3.9	NA

Values are mean ± SD

*VO*_*2max*_ maximal oxygen consumption

The strength training program consisted of two sessions per week and lasted for 11 weeks. Physical tests were conducted before and after the intervention period, and were done over three test days, with similar set-ups before and after the intervention. At the first test-day, leg_LM_ was determined using dual-energy X-ray absorptiometry (DXA). At the second test day, maximal muscle strength was assessed as one-legged 1RM leg press. At the third test day, maximal jump height in CMJ and SJ was measured followed by a maximal isometric torque (MVC) and an isokinetic peak torque test at 240°·sec^−1^ in knee extension. For each participant, all tests were performed at the same time of day before and after the intervention (± 1 h). Prior to physical tests at pre-intervention, participants were given a supervised familiarization session. In this session, proper lifting technique and execution were practiced in all test, and individual equipment settings were found.

### Training

Strength training was performed as described in Vikmoen et al (2016a). Briefly, each training session consisted of half squat in a smith machine, leg press with one leg at a time, standing one-legged hip flexion, and ankle plantar flexion. An investigator supervised all workouts during the first 2 weeks, and at least one workout per week thereafter. During the first 3 weeks, participants trained with 10RM sets at the first session and 6RM sets at the second session of the week. These alternating loads were adjusted to 8RM and 5RM in weeks 4–6, and were further adjusted to 6RM and 4RM in weeks 7–11. The number of sets in each exercise was always three. During warm-up to every training session, participants ingested a protein bar containing 15 g of protein and 22 g of carbohydrate (Squeezy recovery bar, Squeezy Sports Nutrition, Braunschweig, Germany). Adherence to the strength training was high, with *E* + *S* athletes completing 21.4 ± 1.0 (range 19–22) and *S* participants completing 21.0 ± 0.8 (range 20–22) of the planned 22 strength training sessions.

### Testing

#### Lean mass in the legs

Leg_LM_ was determined using DXA using Lunar Prodigy densiometer (Prodigy Advance PA + 302047, Lunar, San Fransisco, CA, USA). Participants were instructed to refrain from training for the 24 h leading up to the measurement and to refrain from ingesting food or liquid for the 2 h preceding the measurement. Data from two participants in *S* were excluded from the data set due to technical problems with analyses. Therefore, the numbers of participants included in the leg_LM_ data are 11 in *E* + *S* and 8 in *S.*

#### 1RM in one-legged leg press

Following 10 min warm-up on a cycle ergometer, the 1RM test started with a specific warm-up, consisting of three sets with gradually increasing load (40, 75 and 85% of expected 1RM) and decreasing number of repetitions (10 → 6 → 3). The first attempt was performed with a load approximately 5% below the expected 1RM. The expected 1RM was deduced from data obtained during the familiarization session. If a lift was successful, the load was increased by approximately 5%. The test was terminated when the participants failed to lift the load in 2–3 attempts. The highest successful load lifted was defined as 1RM. Participants were given 3 min of rest between lifts.

#### SJ and CMJ

After 10-min warm-up on a cycle ergometer, SJ and CMJ jumps were performed on a force plate (SG-9, Advanced Mechanical Technologies, Newton, MA, USA, sampling frequency of 1 kHz). After 3–5 submaximal warm-up jumps, the participants performed three SJ and three CMJ with 2–3 min rest between each jump. The mean of the two highest SJ and CMJ were utilized for statistical analyses. During all jumps, the participants were instructed to keep their hands placed on their hips and to aim for maximal jumping height. The SJ was performed from approximately 90 degrees knee angle. In this position, they paused for 3 s before the jump was performed. No downward movement was allowed prior to the jump and the force curves were inspected to verify this. During the eccentric phase of the CMJ, the participants were instructed to turn at a knee angle they felt was optimal for achieving maximal jump height. Vertical jumping height was calculated from the impulse from the ground reaction force.

#### Maximal isometric torque and isokinetic torque at an angular velocity of 240°·sec^−1^

After the jump tests, peak torque during MVC and isokinetic knee extension were measured in a dynamometer (Cybex 6000, Cybex International, Medway, USA). During these tests, the participants were seated with a 90° hip angle and were stabilized in this position using chest, hip and thigh straps. The input axis of the dynamometer was aligned with the participants’ knee joint and the ankle was strapped to a lever arm. The participants held their arms in front of their chest during all tests. First, the participants performed three maximal knee extensions against the lever arm at a 90° knee angle. Contractions lasted for 5 s, with 1 min rest between attempts. The participants were instructed to perform the muscle action as forcefully and quickly as possible. The attempt with the highest maximal torque was chosen for statistical analyses. Two minutes after the last MVC, three maximal isotonic knee extensions were performed from 90° knee angle to full extension against the lever arm at an angular velocity of 240°·sec^−1^, with one minute rest between attempts. The attempt with the highest torque was chosen for statistical analyses. Strong verbal encouragement was given to participants during all attempts but without live visual feedback of the torque curves.

### Dietary intake

In the sixth training week, participants recorded their daily dietary intake for four days using the weighed-food-intake method. These 4 days included 3 weekdays (not Friday) and either Saturday or Sunday. The participants weighed all their consumed food using digital food weights (Wilfa KW-4, Wilfa AS, Hagan, Norway) and tracked their intake in written journals. All participants were given detailed written and verbal guidelines about how to carry out this method. Dietary data were analyzed using a nutrient analysis software (Kostholdsplanleggeren 2014, Norwegian Food Safety Authority and The Norwegian Directorate of Health, Oslo, Norway).

### Statistics

All data in the text, figures and tables are presented as mean ± standard deviation, unless otherwise stated.

Unpaired Student’s *t* test were utilized to test for differences between groups at pre and post, and to test for differences in changes from pre to post between groups. Paired *t* tests were utilized to test for within-group analyses. Effect sizes (ES) were calculated for key performance and physiological adaptations between groups to elucidate on the practical significance of strength training. ES were calculated as Cohen’s d and the criteria to interpret the magnitude were the following: 0–0.2 = trivial, 0.2–0.6 = small, 0.6–1.2 = moderate, 1.2–2.0 = large and ˃ 2 = very large (Hopkins et al. 2009).

## Results

### Lean mass in the legs_,_ training load and maximal strength

**Fig. 1:**
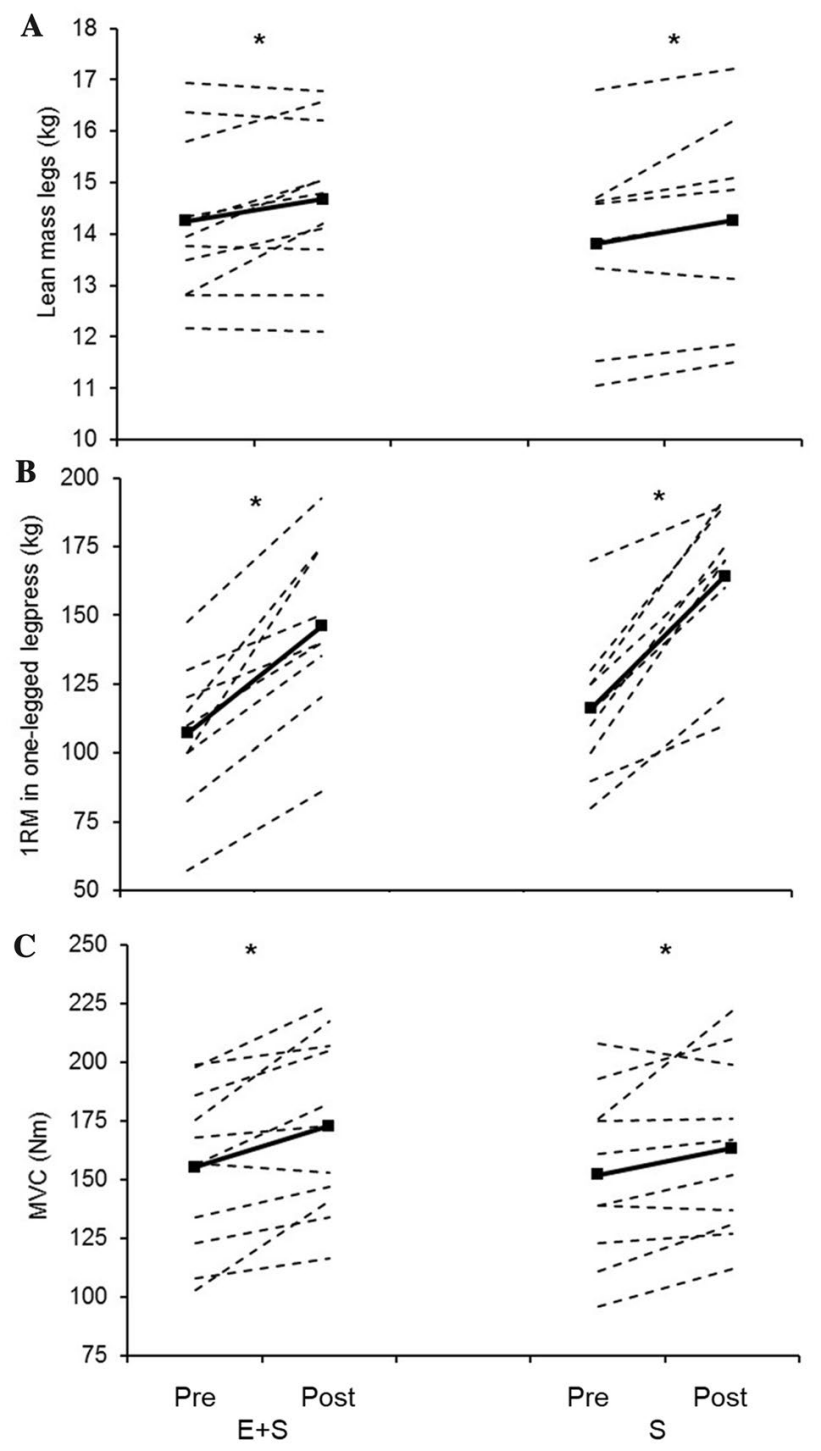
Individual values (dotted lines) and mean values (solid lines) before (pre) and after (post) the intervention period for athletes adding strength training to their normal endurance training (*E* + *S*), and previously untrained individuals performing strength training only (*S*). **a** Lean mass in the legs. **b** 1 repetition maximum (1RM) in one-legged leg press. **c** Maximal isometric torque in knee extension (MVC). *Larger than pre (*p* < 0.05)

**Fig. 2:**
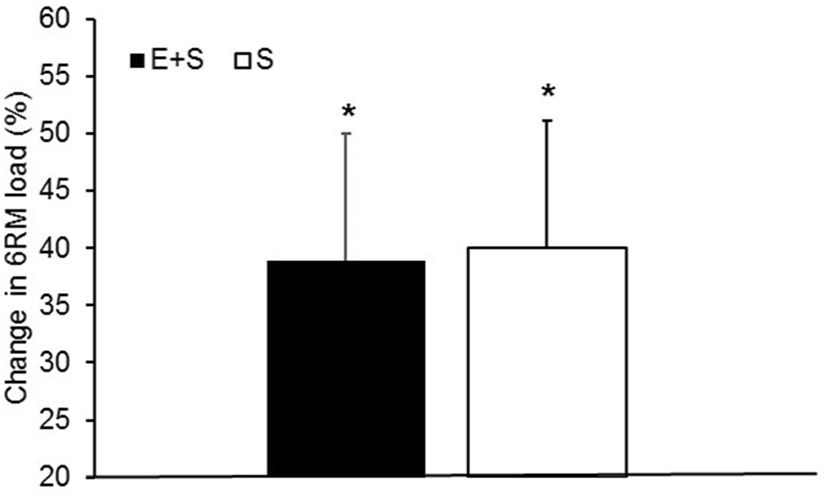
Percent change in 6 repetition maximum (6RM) load from training week 2 to training week 11 during the intervention period for athletes adding strength training to their normal endurance training (*E* + *S*), and previously untrained participants performing strength training only (*S*). *Significant increase from pre (*p* < 0.01)

Body mass remained unchanged in *E* + *S* (from 62.4 ± 5.2 to 63.1 ± 5.6 kg) and in *S* (from 67.8 ± 13.5 to 68.0 ± 12.3 kg). In *E* + *S* and *S*, 11 weeks of strength training led to similar increases in Leg_LM_ (*E* + *S* 3.1 ± 4.0%, *S* 3.3 ± 3.3%, *p* ˂ 0.05; Fig. 1), 1RM one-legged leg press (*E* + *S* 39 ± 19%, *p* ˂ 0.05, *S* 42 ± 17%, *p* ˂ 0.05; Fig. 1), maximal isometric torque (*E* + *S* 12 ± 11%, *S* 8 ± 10%, *p* ˂ 0.05; Fig. 1), and progression in 6RM load (kg) from week 2 to week 11 (*E* + *S* 39 ± 11%, *S* 40 ± 11%, *p* ˂ 0.05; Fig. 2).

### SJ and CMJ

**Fig. 3:**
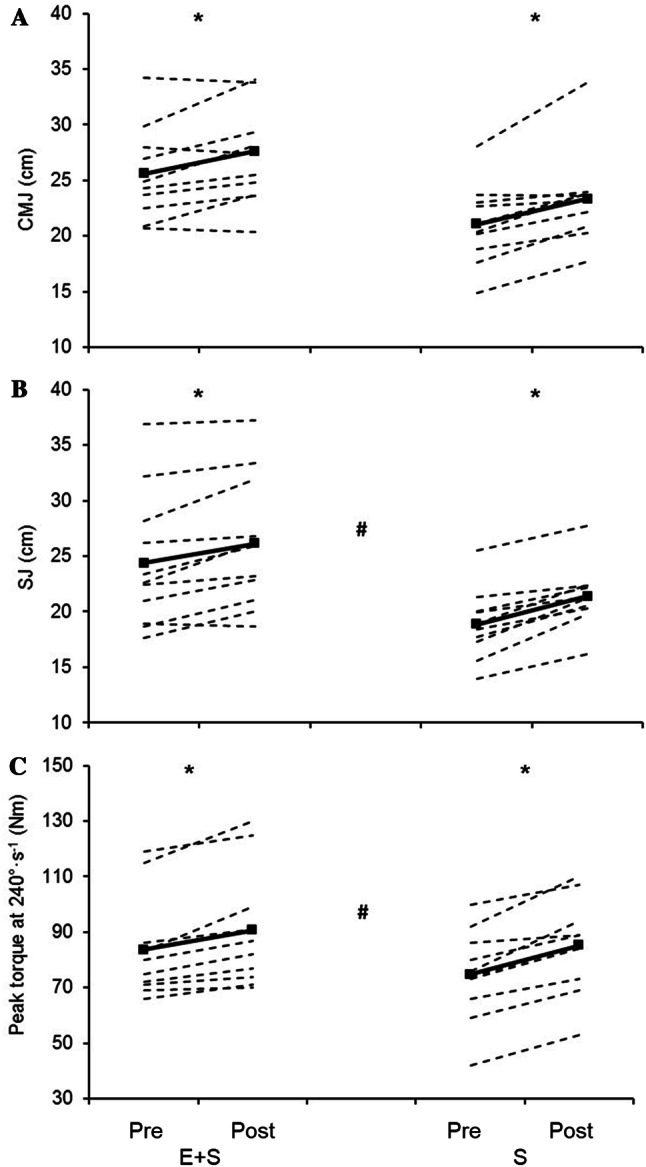
Individual values (dotted lines) and mean values (solid lines) before (Pre) and after (Post) the intervention period for athletes adding strength training to their normal endurance training (*E* + *S*), and previously untrained individuals performing strength training only (*S*). **a** Counter movement jump (CMJ). **b** Squat jump (SJ). **c** Peak torque in isokinetic knee extension at an angular velocity of 240°·s^−1^. *Larger than pre (*p* < 0.05), # the percent change from pre is different between *E* + *S* and *E* (*p* < 0.05)

Before the intervention period, *E* + *S* performed better than *S* (Fig. 3) in both SJ (*E* + *S* 24.3 ± 6.0 cm, *S* 18.9 ± 3.2 cm, *p* ˂ 0.05) and CMJ (*E* + *S* 25.6 ± 4.2 cm, *S* 21.0 ± 3.6 cm, *p* ˂ 0.05).

The intervention led to improved SJ (*E* + *S* 8 ± 6%, S 14 ± 7%, *p* ˂ 0.05) and CMJ (*E* + *S* 6 ± 6%, *S* 11 ± 8%, *p* ˂ 0.05) in both groups (Fig. 3). The increase in SJ was larger in *S* than in *E* + *S* (*p* ˂ 0.05) while there was no statistically difference in the increase in CMJ (*p* = 0.11). The effect size analyses revealed a moderate practical effect in favor of the *S* group for both SJ (ES = 0.95) and CMJ (ES = 0.73). Peak torque at 240°·sec^−1^ was improved in both groups (*E* + *S* 8 ± 5%, *S* 15 ± 7%, *p* ˂ 0.05, Fig. 3). The improvement in *S* was significantly larger than in *E* + *S* (*p* ˂ 0.05, Fig. 3), with a moderate practical effect (ES = 1.11).

### Dietary intake

There was no difference in total energy intake or carbohydrate and fat intake between *E* + *S* and *S,* neither in absolute values nor in values normalized to body mass (Table 2). However, *E* + *S* had higher protein intake than *S,* both in absolute values (*p* ˂ 0.05, Table 2) and as values normalized to body mass (*p* ˂ 0.05, Table 2).

**Table 2:** Energy and macro-nutrient intake for athletes adding strength training to their normal endurance training (*E* + *S*) and previously untrained individuals performing strength training only (*S*)

Nutrient	*E* + *S*	*S*
Energy intake (kJ day^−1^)	8901 ± 2119	7752 ± 811
Energy intake (kJ kg^−1^ day^−1^)	141 ± 24	123 ± 32
Carbohydrate (g day^−1^)	218 ± 93	232 ± 35
Carbohydrate (g kg^−1^ day^−1^)	3.5 ± 1.5	3.6 ± 0.9
Protein (g day^−1^)	104 ± 26*	67 ± 26
Protein (g kg^−1^ day^−1^)	1.7 ± 0.4*	1.2 ± 0.3
Fat (g day^−1^)	80 ± 26	75 ± 11
Fat (g kg^−1^ day^−1^)	1.3 ± 0.3	1.2 ± 0.3

Values are mean ± SD

*Larger than *S* (*p* < 0.05)

## Discussion

In the current study, 11 weeks of strength training led to similar improvements in leg muscle mass and development of force at low contraction velocities in endurance athletes maintaining their normal endurance training and untrained individuals. Interestingly, the ability to develop muscular power increased more in the untrained participants as shown by the greater improvement in jumping performance and maximal isokinetic torque at high contraction velocities.

### The ability to develop force at low contraction velocities and leg_LM_

In the present study, concurrent training did not impair adaptation in the ability to develop force at low contraction velocities and muscle hypertrophy, as evident from similar increases in 1RM, MVC and leg_LM_ between *E* + *S* and *S*. This contradicts the interference effect, a phenomenon that was first reported by Hickson (1980), where concurrent training led to smaller increases in 1RM squat during the last 3 weeks of a 10-week training period compared to a group performing strength training only. The interference effect has since been supported by several studies demonstrating attenuated changes in maximal strength (Bell et al. 2000; Cadore et al. 2010; Gergley 2009; Hickson 1980; Hunter et al. 1987; Izquierdo et al. 2005; Jones et al. 2013; Kraemer et al. 1995) and muscle hypertrophy (Bell et al. 2000; Karavirta et al. 2011; Kraemer et al. 1995; Putman et al. 2004; Ronnestad et al. 2012). However, there are also numerous studies reporting no negative effect of concurrent endurance training on changes in maximal strength at low contraction velocities and muscle hypertrophy after a strength training intervention (Abernethy and Quigley 1993; Cantrell et al. 2014; Holviala et al. 2012; McCarthy et al. 1995, 2002; Nelson et al. 1990; Shaw et al. 2009). The reasons for these conflicting results are unclear, but they are probably due to methodical differences between studies, for example in training status of study participants and total training volume.

Studies that report impaired increases in maximal strength and muscle hypertrophy in response to concurrent training tend to include higher numbers of total training sessions (six or more) and endurance training sessions per week (Bell et al. 2000; Hickson 1980; Hunter et al. 1987; Jones et al. 2013; Kraemer et al. 1995) than studies that do not report attenuated adaptations (Holviala et al. 2012; McCarthy et al. 1995, 2002; Shaw et al. 2009), even though exceptions exist (de Souza et al. 2007; Gergley 2009; Izquierdo et al. 2005). Therefore, the total amount of concurrent training performed is probably an important factor for impairments in strength training adaptations to occur. In accordance with this, Jones et al. (2013) reported attenuated strength adaptations in recreationally strength-trained men after concurrent training with three endurance training session per week but not with one session.

The present study investigated whether well-trained female endurance athletes, maintaining a steady level of endurance training, show different adaptations to strength training than previously untrained individuals. With this design, the actual increase in training volume from adding strength training was similar between intervention groups. Our results conflict with data from a previous study using a similar design (Ronnestad et al. 2012), where increases in 1RM and muscle CSA were attenuated in well-trained male cyclists performing 12 weeks of heavy strength training compared to recreational active individuals performing strength training only. This discrepancy may be due to the amount of endurance training performed. In the current study, *E* + *S* performed about 5 h of endurance training per week, as compared to about 10 h per week in the study by Ronnestad et al. (2012). Supporting this, in recreationally active runners, 1–3 h of endurance training did not impair strength training adaptations (Hunter et al. 1987). Although the large differences in endurance training volume seems like a plausible explanation for why we, in contrast to Ronnestad et al. (2012), did not find attenuated muscle hypertrophy with concurrent training, we cannot exclude possible sex differences.

The higher protein intake in *E* + *S* compared to *S* poses a challenge for interpretation of the changes in leg_LM_ in the two groups. Nutritional status, especially energy balance and protein intake, impacts muscular adaptations to strength training (Garthe et al. 2013; Rodriguez et al. 2009), with protein intake alone also stimulating myofibrillar protein synthesis (Phillips et al. 2005). A recent meta-analysis concluded that protein supplementation up to a daily intake of 1.62 g/kg augments strength training-induced gains in muscle mass (Morton et al. 2018). The *S* group in the current study was below this limit. However, both groups had protein intakes that were within ACSM’s recommendations for endurance- and strength-trained athletes (Rodriguez et al. 2009). Moreover, the overall energy intake was similar between groups, despite *E* + *S* performing 5 h of endurance training per week indicating that the total energy balance was more positive in *S* than in *E* + *S*. Therefore, it is difficult to assess the effects of the different protein intake between the groups, but we cannot exclude the possibility that the larger protein intake in *E* + *S* may have mitigated a possible negative effect of endurance training on the increase in leg_LM_.

### The ability to develop forces at high contraction velocities and jumping ability

In *E* + *S*, strength training-associated increases in peak torque at high contraction velocities and jumping performance was attenuated. Concurrent training thus impaired power-related adaptations, without affecting increases in force production at low muscle contraction velocities or hypertrophy, largely resembling some previous classic concurrent training studies (Dudley and Djamil 1985; Glowacki et al. 2004; Hakkinen et al. 2003). Attenuated increase in jumping performance after strength training have also been reported in well-trained male cyclists continuing their normal endurance training (Ronnestad et al. 2012).

The underlying mechanisms behind concurrent training negatively affecting power-related adaptations more than maximal strength and hypertrophy remain unclear, and our study design was not suited for elucidating on this perspective. However, the ability to produce power and force at high contraction velocities is in addition to maximal muscular strength, depended on relative proportions of type II muscle fibers (Fitts and Widrick 1996), rapid neural activation of the muscles (Folland and Williams 2007; Rhea et al. 2008) and muscle fascicle length (Blazevich 2006). Concurrent training does not seem to affect strength training-induced adaptations in type II muscle fibers (Kraemer et al. 1995; Putman et al. 2004), at least not in subjects with similar training status. In addition, studies reporting impaired hypertrophy in muscle fibers after concurrent training indicate that this happens predominately in the type I fibers (Bell et al. 2000; Kraemer et al. 1995). Therefore, the effects of concurrent training on fiber type do probably not explain the impairing effects on power and rapid force production. Hakkinen et al. (2003) reported reduced adaptations in RFD together with a lack of increased iEMG in *m. vastus lateralis* muscle during the first 500 ms of isometric knee extension after concurrent training compared to strength training only, indicating attenuated development of rapid voluntary neural activation (Hakkinen et al. 2003). It remains unknown if concurrent training affects muscle fascicle length differently than strength training alone. To our best knowledge, no concurrent training study has investigated this aspect and studies investigating the effects of endurance training on muscle architecture yields diverging results (Farup et al. 2012; Murach et al. 2015).

Notably, the superior gains in jump performance in S in the present data could be the result of lower performance levels at baseline compared to *E* + *S*, perhaps related to better motor skills and non-significant lower body mass. As the two groups performed similarly in all other strength exercises at baseline, they seemed to possess similar abilities to activate slow and fast muscle fibers. Furthermore, the smaller increase seen in in peak torque at high contraction velocities in the less coordinative demanding knee extension exercise, supports that the superior gains in jump performance in S was because of an interfering effect of concurrent training in *E* + *S.* It thus seems reasonable to suggest that the difference in improvement in jumping performance between *E* + *S* and *S* was related to the concurrent training protocol.

## Conclusion

In the present study, well-trained female endurance athletes who maintained a steady-state endurance training consisting of about 5 h per week had similar increases in the ability to develop force at low muscular contractions velocities, and comparable gains in leg_LM_ after 11 weeks of heavy strength training as previously untrained females. However, concurrent training attenuated strength training-associated changes in development of force at higher muscular contraction velocities. This supports the notion that concurrent training interferes with power-related adaptations to strength training.

## Abbreviations

1RM: One repetition maximum
CMJ: Counter movement jump
DXA: Dual-energy X-ray absorptiometry
ES: Effect size
E + S: Endurance plus strength training group
Leg_LM_: Lean mass in the legs
MVC: Maximal isometric torque
RFD: Rate of force development
S: Strength training only group
SD: Standard deviation
SJ: Squat jump
